# Long-Term Follow-up of Unilateral Cleft lip and Palate: Incidence of Speech-Correcting Surgeries and Fistula Formation

**DOI:** 10.1177/10556656211055641

**Published:** 2021-12-06

**Authors:** Charlotta Gustafsson, Arja Heliövaara, Junnu Leikola

**Affiliations:** Cleft Palate and Craniofacial Center, Helsinki University and Helsinki University Hospital, Helsinki, Finland

**Keywords:** velopharyngeal function, nonsyndromic clefting, palatoplasty, pharyngoplasty, bone grafting, surgical technique

## Abstract

**Objective:**

The ideal surgical protocol and technique for primary closure of unilateral cleft lip and palate (UCLP) are unclear, and the development of velopharyngeal insufficiency and fistulae following primary repair is common. This study aimed to determine the long-term surgical burden of care in terms of secondary surgeries, defined as speech-correcting surgeries (SCSs) and fistula repair, in a UCLP population, and to compare outcomes of various surgical protocols.

**Design:**

Retrospective, single-center review.

**Participants:**

The study comprised 290 nonsyndromic children with complete UCLP. Different surgical protocols entailing both single-stage and 2-stage approaches were compared, and the surgical outcome was analyzed at the time of alveolar bone grafting (ABG) and post ABG.

**Results:**

Altogether 110 children (37.9%) underwent secondary surgery by the time of ABG. Of the total population 25.9% (n  =  75) had undergone SCS and 17.2% (n  =  50) had undergone fistula repair. The respective incidences at follow-up (post ABG) were 30.3% (n  =  88) and 18.9% (n  =  55). Median age at ABG was 9.8 years and at follow-up was 16.3 years. No significant difference emerged in terms of secondary surgeries between the techniques and protocols applied at primary repair. However, some differences occurred regarding the location of fistulae; the single-stage procedure had more anterior fistula repairs, particularly connected to a perialveolar fistula.

**Conclusions:**

Although the outcome differences between the surgical protocols were small, indicating that none of the treatment protocols was clearly superior to another, attention was drawn to the favorable outcomes of the single-stage protocol.

## Introduction

Protocols of care for the management of individuals affected by cleft lip and palate include both nonsurgical and surgical interventions. The goal of primary cleft surgery is to achieve a normal appearance, hearing, and velopharyngeal function allowing normal development of speech, without restricting the growth of the maxilla (Agrawal, 2009). As such, numerous surgical protocols and surgical techniques have been outlined in the literature (Semb et al., 2017; Reddy et al., 2018; Rossell-Perry, 2018). However, while there is still no single accepted protocol for the surgical treatment for patients with cleft lip and palate in terms of both the type of surgical procedure conducted and the timing of that procedure, the vast majority of primary cleft surgery is typically completed by the first year of age. The need for secondary surgeries of the palate is generally considered a quality measurement of the primary surgery (Tache and Mommaerts, 2019).

Postoperative velopharyngeal insufficiency (VPI) is common in children with a cleft and VPI might result in detrimental outcomes for the child, often requiring speech-correcting surgeries (SCSs) (VPI surgery) if conservative management, in terms of speech therapy, is inadequate (Becker et al., 2004). However, several factors may influence the rate of SCS. Such as the severity of the VPI, the family’s wishes, associated anomalies, airway obstruction, the speech-language pathologist’s recommendations, but also the surgeon’s experience and threshold to perform secondary surgery have a major role. Development of palatal fistulae, caused by improper healing of the palate, is another well-known and undesired complication following primary repair. Symptomatic palatal fistulae may cause nasal regurgitation and have an adverse effect on speech, necessitating surgical intervention (Hardwicke et al., 2014).

The success of primary surgery is commonly measured by speech-related outcomes and fistula occurrence following cleft repair (Landheer et al., 2010; Reddy et al., 2018; Rossell-Perry, 2018; Stein et al., 2019), while the definite operative burden often remains unclear. This study aimed to report the surgical burden of care in terms of secondary surgeries, defined as SCS and fistula closure, on a long-term perspective, up to alveolar bone grafting (ABG) and beyond, in patients with complete unilateral cleft lip and palate (UCLP) treated at a single institution. Our secondary aim was to assess the surgical protocols and techniques utilized over the years within the unit to examine their impact on secondary surgery rates.

## Methods

### Study Design and Patients

We conducted a retrospective, single-center review of clinical records of patients with UCLP treated at the Cleft Palate and Craniofacial Center of Helsinki University Hospital, Finland. The primary objectives were to determine long-term incidence for SCS and repair rates for postalveolar fistulae. The secondary objective was to compare the different surgical protocols and techniques used over the past few decades at the cleft center.

The study protocol was approved by Helsinki University Hospital. Principles outlined in the Declaration of Helsinki were followed. The patients were born between 1990 and 2011. Patients with incomplete clefts of the lip and palate, inadequate clinical data, unperformed ABG, or other comorbidities, such as syndromes and other craniofacial anomalies in the head or neck region, were excluded. However, patients with Simonart’s bands were included in the study.

The following data were extracted from medical records: date of birth, gender, birth weight and length, gestational age, total follow-up time, surgical technique used, date of primary lip repair, and primary palatoplasty. If performed, the age and technique used at SCS and fistula closure and the location of the repaired fistula were also recorded.

Data were analyzed at the time of the secondary ABG since a substantial part of the secondary surgeries, particularly fistula closures, are performed at this time point, and hence, it gives a more precise picture of the specific time points at fistula repair. At our center, secondary ABG (with cancellous bone from iliac crest) is typically performed between 9 and 11 years of age, before eruption of the upper canines, while the majority of SCS are performed earlier. To achieve an even broader long-term picture, we also analyzed the outcomes after ABG (post ABG); until the end of the patients’ follow-up, or at the last follow-up, prior to data collection (January 2021).

### Classification of Primary Surgery

While primary nasal repair has for decades generally been performed alongside lip repair via the principles described by McComb (1975), the protocol for primary lip and palate surgery (both age at surgery and surgical technique) have changed over the last 3 decades within our cleft center. This has been influenced by a number of factors including the unit’s participation in the Scandcleft randomized control trials.

Early in the 1990s, cleft lip and palate was repaired by lip adhesion at 2 months of age, followed by cheiloplasty and anterior vomerplasty at 6 months, with complete cleft closure at 12 months of age according to the Veau–Wardill–Kilner (V–W–K) technique (Dorrance and Bransfield, 1946), which had been the standard technique for decades.

Lip adhesion was later considered unnecessary; it was therefore discarded, as was anterior vomerplasty. Simultaneously, lip closure was altered to an age of 3 to 4 months and performed by the chileoplasty technique described by Milliard (1958), which has since retained its place as the standard technique. In 1992, the standard technique became the 2-flap approach described by Bardach (1995), where the palate closure took place at 9 months of age in combination with a vomer flap. As of 1994, the 2-flap approach was the standard procedure for particularly wide and challenging clefts, while the minimal incision technique, outlined by Mendoza et al. (1994) was employed for muscle reconstruction of narrower clefts; if tension occurred, lateral relaxing incisions were made according to the von Langenbeck technique (von Langenbeck, 1861).

In 1996, slightly prior to the recruitment of patients for the Scandcleft randomized trials of primary surgery for UCLP (Rautio et al., 2017), the 2-stage closure was introduced at our center, where lip and soft palate repair occurred at 4 months combined with delayed hard palate closure at 12 months. The hard palate was closed using a vomer flap, while the muscle repair was performed according to the principles described by Sommerlad (2003). When the Scandcleft Trail2 (1997-2006) began, half the patients received the 2-stage procedure just described and the other half of the patients received a single-stage palatoplasty at 12 months by a common technique, such as the 2-flap, minimal incision, or Langenbeck’s technique, in combination with a vomer flap. In 2008, the 2-stage protocol was further altered into combined lip and early hard palate closure, with a vomer flap, at 4 months with delayed soft palate closure at ∼10 months. This procedure has since remained as the main protocol at our center.

### Classification of SCSs

Patients’ speech was repeatedly assessed by a multidisciplinary cleft team during frequent follow-up visits according to the standard protocol of the cleft center. Speech was assessed by experienced speech-language pathologists via protocols described by Gustafsson et al. (2018). SCS was suggested if the child had significant VPI and presented insufficient improvement after speech therapy. The surgical techniques utilized were pharyngeal flaps and Furlow Z-plasty (1986).

### Classification of Fistulae

The description of palatal fistulae varies greatly in the literature from asymptomatic small fistulae to large symptomatic fistulae that more often require surgical repair. To elucidate the ambiguity among intentional perialveolar fistulae and unintentional fistulae in the secondary palate, standardized classification systems (Smith et al., 2007) have been proposed. However, major inconsistencies in terminology still occur, hindering the drawing of definite conclusions. In addition, widely ranging follow-up times cause underreporting of fistulae. Hence, specific time points in fistula reporting have been recommended (Shankar et al., 2018). While some authors choose to only report symptomatic fistulae requiring surgical intervention to avoid reporting bias (Rautio et al., 2017).

**Figure 1.**
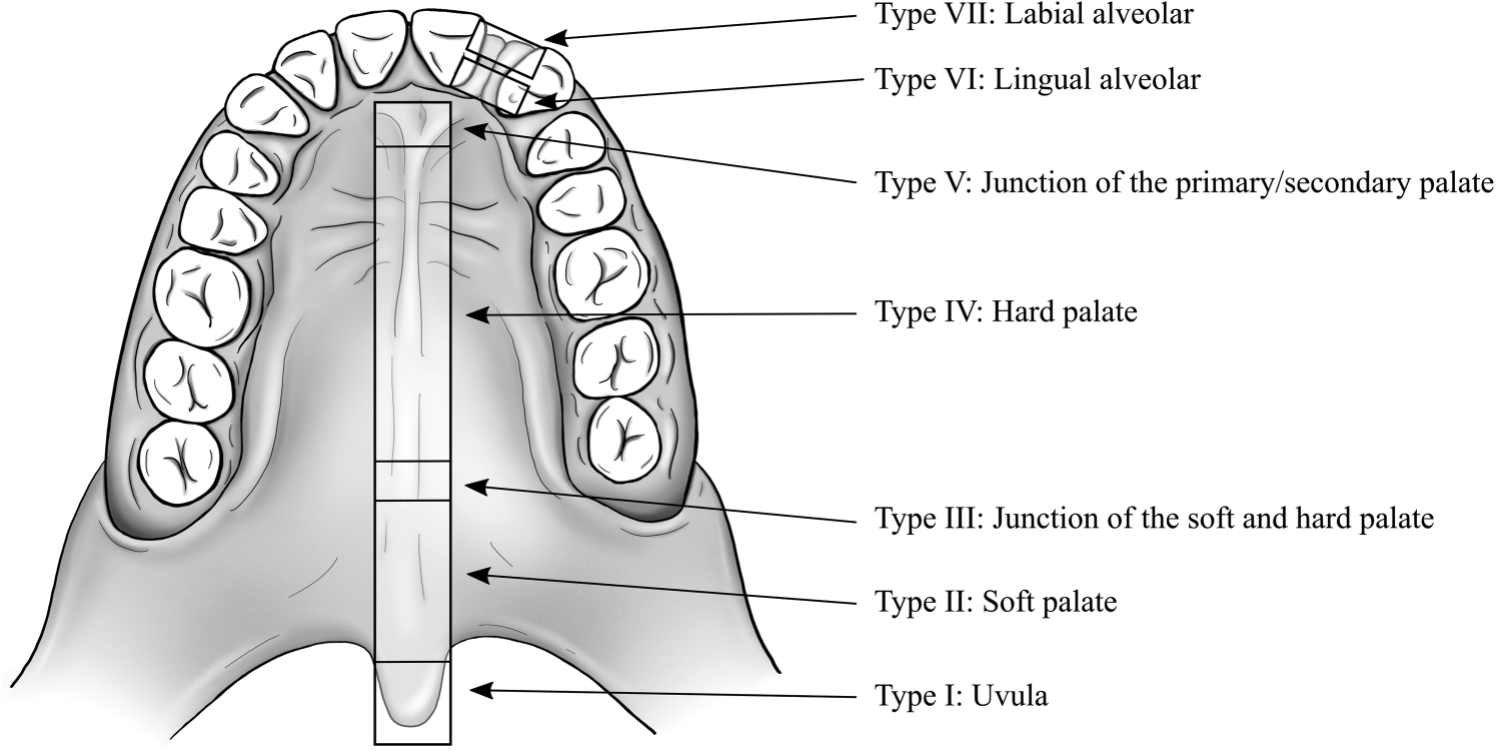
The Pittsburgh fistula classification system according to Smith et al. (2007).

In this study, a fistula in the secondary palate was considered an unintended defect in the corrected palate resulting from improper healing. Due to the limitations posed by the retrospective nature of this study and the lack of standardized methodologies to discern palatal fistulae, we decided to only review fistulae that had received a clinical recommendation for surgical closure. As such, the precise location and nature of the fistula were identified from the operative records. However, it is possible that some patients presented clinically with asymptomatic fistulae. The location of the palatal fistula was classified according to the Pittsburgh classification system (Smith et al., 2007) (Figure 1). Fistulae anterior to the incisive foramen were considered lingual/labial-alveolar fistulae (Pittsburgh VI and VII); such perialveolar fistulae are often intentionally left open at primary palate repair.

If several fistulae were repaired in the palate at different locations, they were counted as separate fistulae. However, if the repaired fistula crossed several Pittsburgh locations, it was counted once, according to its most anterior location.

The technique for fistula repair differed among surgeons, but mainly through suturing the oral and nasal mucosa in separate layers, with the potential use of lateral incisions, and resorbable collagen membrane.

### Statistical Analysis

All analyses were performed with SPSS Statistics for Macintosh, Version 25.0 (IBM Corp.). Categorical comparisons were made with Pearson’s chi-squared test and Fisher’s exact test and the results are presented as proportions (%). A comparison of continuous nonparametric variables was performed with Mann–Whitney *U* and Kruskal–Wallis tests, and the results are presented as medians with interquartile ranges (IQRs). Differences were considered significant when *P* < .05.

## Results

### Patients

After reviewing 438 files of patients with UCLP, 148 were excluded because they did not meet the inclusion criteria. This analysis is based on the remaining 290 patients (male n  =  171; 58.9%) of which 20.7% (n  =  60) presented with Simonart’s band. The median age at ABG was 9.8 (9.3-10.5) years, and the median total follow-up (post ABG) age at data retrieval was 16.3 (12.0-19.1) years (Table 1).

**Table 1.** Population Demographics.

	Single-stage	Two-stage		
		Delayed HCP repair	Early HCP repair	* P*
No. of patients	186	63	41	
Gender (male)	105 (56.8)	40 (63.5)	26 (63.4)	.538
Cleft side (left)	107 (57.5)	41 (65.1)	26 (63.4)	.509
Birth weight, grams	3480 (3102.5-3995.0)	3625 (3315.0-3995.0)	3570 (3147.5-3977.5)	.160
Gestational age, weeks	40 (39.0-40.3)	40 (39.0-41.0)	40 (39.0-41.0)	.208
Age at lip repair, months	3.9 (3.4-4.7)	3.9 (3.7-4.2)	4.0 (3.6-4.3)	.821
Age at first palatoplasty, months	10.9 (9.6-12.2)	3.9 (3.7-4.2)	4.0 (3.6-4.3)	<.001
Age at second palatoplasty, months	N/A	12.1 (11.6-12.6)	10.1 (9.3-11.4)	<.001
Age at ABG, years	9.6 (9.1-10.4)	9.9 (9.5-10.5)	10.1 (9.6-10.8)	.008
Post ABG, total follow-up time, years	17.4 (13.1-20.4)	17.0 (14.3-18.4)	10.5 (10.0-11.6)	<.001

Abbreviations: IQR, interquartile range; ABG, alveolar bone transfer; HCP, hard cleft palate; Post ABG, total follow-up time at data collection.

Categorical data presented as n (%), continuous data as median (IQR).

### Surgical Protocol

Within the last few decades, both single-stage and 2-stage approaches have been performed at the cleft center. The majority (64.1%; n  =  186) of patients have received primary closure in a single-stage procedure, with a median age of lip repair at 3.9 months and palatoplasty at 10.9 months. Forty-eight patients of the 1-stage protocol were originally included in the Scandcleft study (Arm C, Trial 2) (Rautio et al., 2017) (see Table 1) and had received 1-stage closure by a common technique at our center. Single-stage repair was performed by various techniques employed at the cleft center over the years (Table 2).

**Table 2.** Incidence for SCSs and Fistula Repair at ABG and Post ABG.

Protocol	No. of patients	SCS (No. of patients (%))				Fistula repair (No. of patients (%))					
		ABG	*P*	Post ABG	*P*	Pre ABG	*P*	ABG	*P*	Post ABG	*P*
**Single-stage**	**186**	**42 (22.5)**	.233^a^	**54 (29.0)**	.797^a^	**11 (5.9)**	.106^a^	**35 (18.8)**	.268^a^	**38 (20.4)**	.380^a^
2-flap	94	14 (14.9)	.064^b^	20 (21.3)	.086^b^	4 (4.3)	.311^b^	17 (18.1)	.256^b^	18 (19.1)	.166^b^
Langenbeck	52	17 (32.7)		21 (40.4)		4 (7.7)		8 (15.4)		8 (15.4)	
Minimal incision	16	5 (31.3)		5 (31.3)		0 (0)		2 (12.5)		3 (18.8)	
V–W–K	24	6 (25.0)		8 (33.3)		3 (12.5)		8 (33.3)		9 (37.5)	
(Arm [C])	48	14 (29.2)		16 (33.3)		2 (4.2)		8 (16.7)		9 (18.8)	
**Delayed HCP repair**	**63**	**20 (31.7)**	.997^c^	**21 (33.3)**	.863^c^	**9 (14.3)**	.495^c^	**11 (17.5)**	.274^c^	**13 (20.6)**	.143^c^
(Arm [A])	39	15 (38.5)		15 (38.5)		7 (17.9)		9 (23.1)		11 (28.2)	
**Early HCP repair**	**41**	**13 (31.7)**		**13 (31.7)**		**4 (9.8)**		**4 (9.8)**		**4 (9.8)**	
Total	290	75 (25.9)		88 (30.3)		24 (8.3)		50 (17.2)		55 (18.9)	

Categorical data presented as n (%). ABG indicates alveolar bone grafting; Arm (C) and Arm (A), patients included in Scandcleft randomized study Trial 2 (Rautio et al., 2017); Pre ABG, fistula repair performed before ABG; Post ABG, SCS and fistula repair at total follow-up (data collection); SCS, speech-correcting surgery; Minimal incision, Mendoza’s technique; 2-flap, Bardach’s technique; V–W–K, Veau–Wardill–Kilner technique.

^a^ Comparison of the 3 surgical protocols.

^b^ Comparison of the single-stage techniques.

^c^ Comparison of the 2-stage protocols.

The 2-stage protocols varied in the timing of hard palate and soft palate closure. Early soft palate repair in combination with chileoplasty, median 3.9 (3.7-4.2) months, with late hard palate repair, median 12.1 (11.6-12.6) months, represented 21.7% (n  =  63) of the total population. Thirty-nine patients of the 2-stage protocol were originally included in the Scandcleft study (Arm A, Trial 2).

As the most recent introduced protocol at the center, 14.1% (n  =  41) received early hard palate repair with a vomer flap, median 4.0 (3.6-4.3) months, combined with delayed soft palate repair, median 10.1 (9.3-11.4) months. As cohorts of the most recent protocol, these patients had significantly shorter total follow-up time (post ABG) (median 10.5 years) than the patients of the older protocols (H[2]  =  75.677, *P*  ≤ .001) (Table 1).

Over the past few decades, several cleft surgeons have practiced at the cleft center. In this study, the primary repairs were performed by 7 surgeons, 2 of whom performed 81.7% (n  =  237) of the surgeries (48%, n  =  140 and 33%, n  =  97).

### Primary Objective

**Figure 2.**
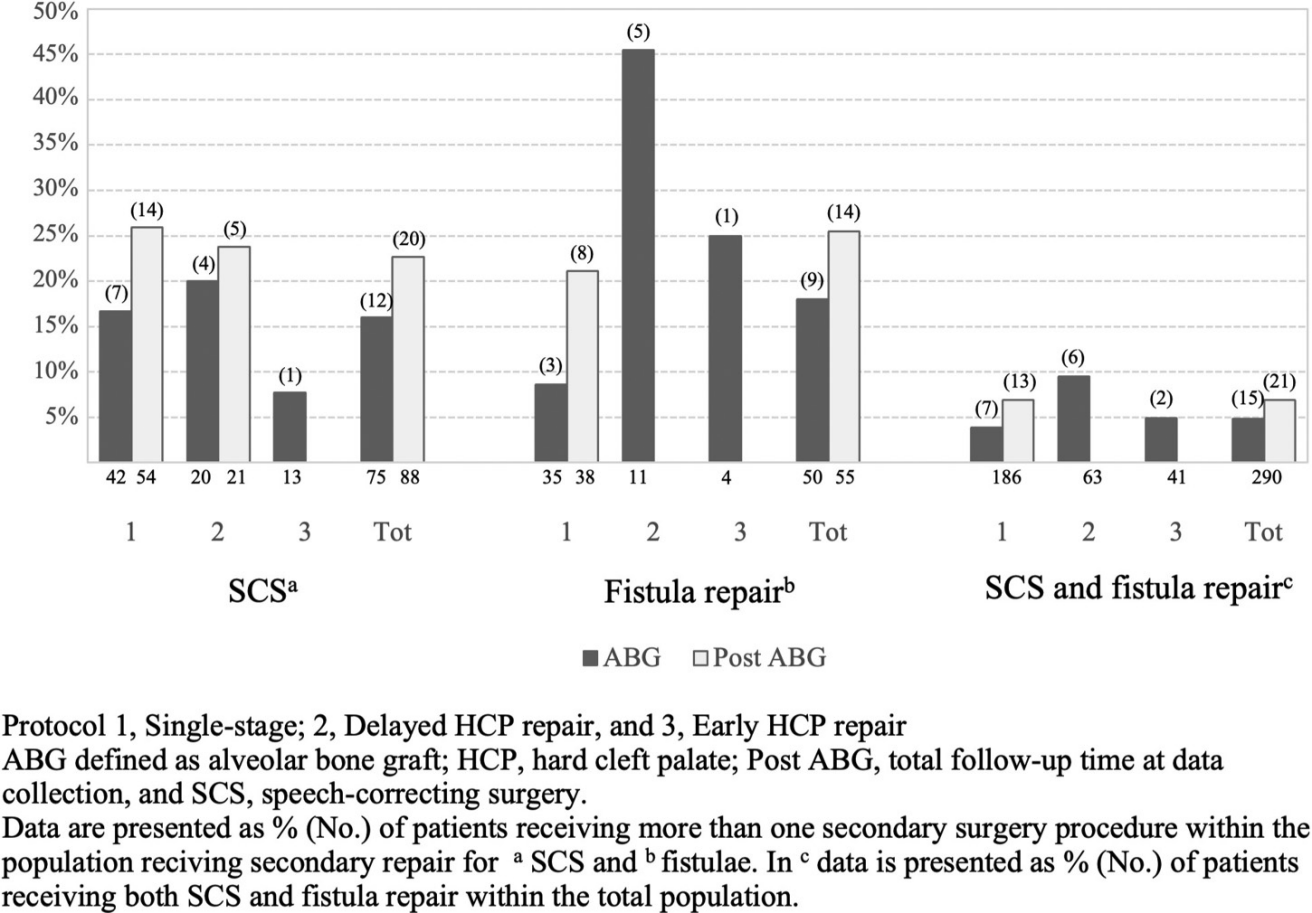
Bar graph of number (%) of patients receiving more than 1 secondary surgery procedure.

By the time of ABG and post ABG, 37.9% (n  =  110) and 42.1% (n  =  122), respectively, underwent secondary surgery. Fifteen patients (5.2%) had received both SCS and fistula repair by the time of ABG, while the respective figure at post ABG was 21 (7.2%) (Figure 2). By the time of ABG, almost one-fourth (23.6%, n  =  26) of those receiving secondary surgery had undergone multiple secondary procedures (including SCS and/or fistula repairs), while the corresponding proportion at post ABG was 30.3% (n  =  37). More specifically 25.9% (n  =  75) had received SCS by the time of ABG, while 17.2% (n  =  50) had received fistula repair. Of these repaired fistulae, 48.0% (n  =  24 of 50) had received repair prior to ABG and 1 fistula was closed in combination with SCS, while the rest were repaired at ABG. By the end of follow-up (post ABG), 30.3% (n  =  88) had SCS and 18.9% (n  =  55) received fistula closure (Table 2). At this time point, 73 (25.2%) had received osteotomy (LeFort 1 osteotomy, n  =  50, and bimaxillary osteotomy, n  =  23) at a median age of 17.1 (15.2-18.9) years due to maxillary hypoplasia. Nine patients (3.2%) had their first SCS performed after osteotomy.

### Speech-Correcting Surgery

No significant difference was noted between the single-stage and the 2 different 2-stage protocols regarding SCS incidence by the time of ABG or post ABG. Similarly, no significant difference emerged between the techniques used in the single-stage approach (Table 2). Yet, when the 2-flap (Bardach) technique was compared with the 2-stage protocols prior to ABG, a significant difference emerged (χ^2^  =  7.961, df  =  2, *P*  =  .019). More specifically, patients operated with the 2-flap technique required only half of the SCS procedures compared with the 2-stage protocols (14.9% vs 31.7%, χ^2^  =  6.314, df  =  1, *P*  =  .012, and 31.7%, χ^2^  =  5.406, df  =  1, *P*  *=*  .020). However, no significant comparison was noted post ABG (χ^2^  =  3.277, df  =  2, *P*  =  .194). The median age for the first SCS prior to ABG was 5.3 (4.4-6.7) years. No significant difference regarding timing of SCS was noted between the surgical protocols. Furlow repalatoplasty was the most often employed SCS technique (72.0%, n  =  54) for VPI by the time of ABG (Table 4). Among patients receiving SCS, 16.0% had 2 or more procedures for VPI by the time of bone grafting, with the corresponding figure at the end of follow-up (post ABG) being 22.7% (Figure 2).

**Table 3.** Location of Repaired Fistulae by Time of ABG.

Protocol	No. of patients	No.of. repaired. fistulas	Pittsburgh location (No. of fistulas (%))				*P*	Connection to perialveolar fistula	*P*
			II	III	IV	V			
**Single-stage**	**186**	**36**	**2 (5.6)**	**3 (8.3)**	**6 (16.7)**	**25 (69.4)**	<.001a	**17 (43.6)**	.006^a.^
2-flap	94	17	1 (5.9)	2 (11.8)	3 (17.6)	11 (64.7)	1.000b	6 (35.3)	.229^b^
Langenbeck	52	8	0	1 (12.5)	1 (12.5)	6 (75.0)		3 (37.5)	
Minimal incision	16	2	0	0	0	2 (100.0)		2 (100.0)	
V–W–K	24	9	1 (11.1)	0	2 (22.2)	6 (66.7)		6 (66.7)	
(Arm [C])	48	8	0	0	1 (12.5)	7 (87.5)		4 (50.0)	
**Delayed HCP repair**	**63**	**13**	**0**	**6 (46.2)**	**6 (46.2)**	**1 (7.7)**	.682c	**N/A**	
(Arm [A])	39	10	0	6 (60.0)	4 (40.0)	0		N/A	
**Early HCP repair**	**41**	**5**	**0**	**2 (40.0)**	**3 (60.0)**	**0**		**N/A**	
Total	290	54	2 (3.8)	11 (20.4)	15 (28.3)	26 (49.1)		17 (32.1)	

Four patients had 2 fistulae repaired at different locations in the palate. No fistula occurred in Pittsburgh location I. Arm (C) and Arm (A) indicates patients included in Scandcleft randomized study Trial 2 (Rautio et al., 2017); Minimal incision, Mendoza’s technique; 2-flap, Bardach’s technique; V–W–K, Veau–Wardill–Kilner technique.

^a^ Comparison of the 3 surgical protocols.

^b^ Comparison of the single-stage techniques.

^c^ Comparison of the 2-stage protocols.

**Table 4.** Surgical Method and age at First SCS.

Protocol	Time point	No. of patients	SCS technique (No. of patients (%))			Age at SCS, years	* P*
			Furlow	Pharyngeal flap	Other		
Single-stage	ABG	42	28 (66.7)	14 (33.3)	N/A	5.3 (4.4-6.1)	.533^a^
	Post ABG	54	38 (70.4)	15 (27.8)	1 (1.9)		
Delayed HCP repair	ABG	20	13 (65.0)	6 (30.0)	1 (5.0)	5.3 (4.5-7.5)	
	Post ABG	21	14 (66.7)	6 (30.0)	1 (4.8)		
Early HCP repair	ABG and Post ABG	13	13 (100.0)	N/A	N/A	5.7 (4.6-6.8)	

Categorical data presented as n (%), continuous data as median (interquartile range [IQR]). ABG indicates alveolar bone transfer; HCP, hard cleft palate; Post ABG, total follow-up time at data collection; SCS, speech-correcting surgery.

^a^ Comparison of the 3 surgical protocols.

A significant difference was noted in SCS rates between the 2 surgeons who had performed the majority (81.7%) of the primary palatoplasties (37.9% vs 14.4%) (χ^2^  =  15.504, df  =  1, *P*  ≤  .001). The surgeon with higher SCS rates performed 90.7% of all SCS that had been done by ABG.

### Repaired Fistulae and Their Location

By the time of ABG and post ABG, no significant difference was noted according to the need for fistula closure between the single- and 2-stage approaches. Although the V–W–K technique led to the highest rate of fistula repair in the single-stage protocol (33% vs 12-18%) (Table 3), the difference was not significant (*P*  =  .256). Similarly, compared to the 2-stage protocols the V–W–K technique had not significantly higher fistula rates (χ^2^  =  5.356, df  =  2, *P*  =  .069). By the time of ABG, the repaired fistulae were most often located in the anterior part of the hard palate (Pittsburgh V, 49.1%, n  =  26) and in the hard palate (Pittsburgh IV, 28.3%, n  =  15). A significant difference was present in the location of the repaired fistulae prior to ABG and at ABG (*P*  =  .002). Prior to ABG, 70.8% (n  =  17) of the repaired fistulae were Pittsburgh IV or V fistulae, while the corresponding proportion at the time of ABG was 92.3% (n  =  24). Similarly, there was a significantly higher incidence of repaired fistulae with connection to a perialveolar fistula at the time of ABG compared to repaired fistulae prior ABG (χ^2^  =  10.793 df  =  1, *P*  =  .001).

The single-stage approach had a high incidence of fistula repair in the anterior part of the hard palate (Pittsburgh V) and was more often (43.6%) associated with a perialveolar fistula than the 2-stage approaches (*P*  =  .006) (Table 3). When comparing the 2-stage approaches, the delayed hard palate closure was associated with fistulae at the border between the hard and soft palate, while early hard palate closure developed more often fistulae in the hard palate. No significant differences emerged between the 2 primary surgeons and fistula repair rates (χ^2^  =  .097, df  =  1, *P*  =  .756)

## Discussion

The optimal surgical protocol in terms of timing and technique in cleft primary repair continues to be debated. Here, our aim was to improve the current information pool by reporting the long-term incidence for secondary surgeries of the palate, comprising SCSs and fistula repair, in a nonsyndromic UCLP population treated at a single cleft center with a history of various surgical protocols and techniques.

There is a general lack of standardized protocols, tools, and techniques for outcome assessment of both speech and fistulae. While there is a general ambiguity in fistula nomenclature (Shankar et al., 2018), speech assessment is a complex process, which generally encompasses perceptual assessment but should also be supplemented by different acoustic and physiologic measures (Kummer, 2014). This limits the drawing of meaningful comparisons between studies and most likely reflects the high reported variations in the literature for VPI and fistula incidence following primary palatal repair (Hardwicke et al., 2014; Shankar et al., 2018; Stein et al., 2019; Yang et al., 2020). The retrospective nature of the vast majority of studies, including comparisons of various Veau clefts with wide differences in treatment protocols, follow-up times, and reporting practices between cleft centers, further challenges the comparison of studies. Most studies concentrate on reporting speech characteristics and identified fistulae following primary repair, while little is reported about the repaired fistulae and VPI surgery rates. However, rates for secondary operations might be a coarse estimate of the success of primary surgery; it is used as a quality measurement for primary repair and more importantly, describes the surgical burden for children with corrected orofacial clefts. Owing to the reasons above, we chose to define initial primary surgical success as the lack of need for secondary surgery in the examination of this patient series.

Distinguishing fistula repair by specific time points highlights the fistula evolvement in the palate. Early fistula repair represents significant fistulae, which develop immediately as a complication following primary palatoplasty, prior to, and during orthodontic treatment and possible palatal expansion. The operative setting during ABG can help to identify true fistulae, which also gives a more precise evaluation of correct fistula incidence in the secondary palate since a significant number of pinhole or small fistulae may go unidentified during the normal examination. Shankars et al. (2018) stressed the importance of reporting time points while examining fistula incidence. Moreover, they reported that 26% of fistulae go undiagnosed before the intraoperative examination during ABG, emphasizing that surgeons must be prepared for fistula repair during ABG, which in turn might increase the operative time. Hence, it may be difficult to interpret the outcome of symptomatic fistula repair at ABG since minor fistulae might be repaired at this time point. This is why some fistula repair is intentionally deferred to this time point.

Various surgical protocols of single- and 2-stage approaches with large differences in the timing of surgery for cleft repair have been described in the literature, yet no consensus exists regarding whether 1 protocol is more favorable than another (Shaw et al., 2001; Rautio et al., 2017). Many centers have well-defined standard protocols, it is not uncommon that surgeons apply a technique by experience, adapting it accordingly to the nature of the cleft. This, in turn, leads to modified techniques, hindering comparisons of particular surgical techniques.

A recent meta-analysis by Stein et al. (2019) reported that single-stage repair is associated with a more favorable outcome concerning VPI and fistula formation relative to a 2-stage repair, which entails early soft palate closure and delayed hard palate closure. Similarly, Ganesh et al. (2015) and Rossell-Perry (2018) found superior surgical outcomes regarding VPI and fistulae with the single-stage procedure compared to the 2-stage approach with early vomer flap repair. In this study, we did not find any significant differences between the 2-stage and single-stage procedures at the long-term follow-up. Our outcomes are in line with an earlier study at our center (Ahti et al., 2020), which assessed velopharyngeal function at 3 and 5 years following primary repair in individuals with UCLP treated with the same protocols. However, although no statistical significance was found, we observed less need for SCS in the single-stage protocol compared with the 2-stage protocols prior to ABG. Such a trend of superior speech outcomes in a single-stage protocol compared to a 2-stage approach is consistent with findings of Stein et al. (2019), Ganesh et al. (2015), and Rossell-Perry (2018). Particularly, patients operated with the 2-flap (Bardach) technique required a low need for SCS and compared with the 2-stage protocols this difference was significantly lower prior to ABG. On the contrary, we observed a high need for fistula repair at ABG in the single-stage approach, particularly in patients that had received primary repair with the V–W–K technique. However, when compared with the other single-stage techniques, as well as with the 2-stage protocols, this finding was not significantly significant. One can speculate whether the repaired palate receives a greater retroposition with the single-stage procedure than with the 2-stage approach, resulting in improved velopharyngeal function with less need for SCS. This supposition is consistent with the findings of Rossell-Perry (2018) who found shorter palatal length in the early vomer flap population compared to the single-stage population, reasoning that an early vomer flap causes scar contracture in the palate.

Consistent with previous studies (Shankar et al., 2018; Yang et al., 2020), we observed a high number of corrected fistulae in the hard palate, especially in the anterior part, at the border to the primary palate (Pittsburgh V). We noted a significant difference in localization of repaired fistulae prior to and at the time of ABG, with a higher prevalence of repaired anterior fistulae in association with a perialveolar fistula at the time of ABG. In terms of the location of the repaired fistulae, the single-stage procedure had a more distributed pattern, with a high representation of anterior fistulae, particularly in association with a perialveolar fistula. The 2-stage procedures, in turn, were linked with fistulae occurring in the hard palate. The delayed hard palate procedure, in particular, was associated with fistula repair at the border of the hard and soft palate. As previously addressed in the Scandcleft study (Rautio et al., 2017), the technical difficulties with constructing the posterior edge of the vomer flap at the border of the soft and hard palate during the second stage of hard palate repair resulted in a tendency toward dehiscence in this region. The high presentation of repaired anterior fistulae in the single-stage procedure is most likely due to the challenge of performing a secure 2-layered closure in the most anterior part of the palate together with the anterior rotation of the palatal shelves during the closure of the cleft that naturally gives rise to a shorter closure anteriorly, resulting in a 1-layered closure, a region susceptible to fistula development. As such, these anterior fistulae, particularly the small ones, are most likely less symptomatic than more posteriorly located fistulae, which often require surgical intervention prior to ABG. While the anterior palatal fistulae are repaired at ABG along with the alveolar fistulae (Pittsburgh VI and VII).

The current protocol at the cleft center, the early hard palate closure with a vomer flap, has been shown to be a reliable technique in closing the hard palate (Martin-Smith et al., 2017). By decreasing the width of the residual cleft, it offers a more favorable condition at delayed soft palate closure (de Jong and Breugem, 2014). This results in reduced fistula development of the palate (Ferdous et al., 2010; Smarius and Breugem, 2016). This trend is also observed in the present study, although no significant difference was noted compared with the 2-stage protocols. However, data on the protocol were rather limited and the follow-up was considerably shorter than in the other protocols. The procedure combined with delayed soft palate repair by the Furlow technique has recently garnered more attention (Liao et al., 2014; Stein et al., 2019), although some concerns regarding the early vomer flap and its subsequent effect on maxillary growth have arisen at our center and elsewhere (Emami and Hashemzadeh, 2020). Further studies are warranted to investigate the impact of the surgical protocol on maxillary growth and dental arch relationships, especially comparing the 1-stage procedure with the early vomer flap.

The strengths of this study include the large and comprehensive data collected at a single center. All patients were treated by an experienced multidisciplinary cleft team with standardized protocols and longitudinal follow-up. Nevertheless, this study has a few noteworthy limitations. The retrospective study design comes with an inherent potential for bias. Surgical techniques are often difficult to compare due to modifications by surgeons in adapting to the severity and anatomy of the cleft. Given the retrospective study design, the authors were constrained by the quality of the operative records. In terms of assessing the localization of palatal fistulae and surgical techniques, deficient and ambiguous records may have resulted in interpretation errors. Unfortunately, we were unable to assess the effect of cleft width on the outcomes, since the measurements were not consistently reported in the medical files, which has earlier been shown to be a prognostic factor for fistula development (Parwaz et al., 2009; Landheer et al., 2010; de Agostino Biella Passos et al., 2014).

As concluded in the Scandcleft study (Shaw and Semb, 2017) surgeons’ experience and skill might outweigh the surgical protocol applied. In the present study, the vast majority of primary surgeries were performed by 2 experienced cleft surgeons. Although our focus was not to analyze the surgeons’ impact on the outcomes, a significant difference was noted between the surgeons and SCS rates, while no difference was noted regarding fistula repair rates. While there may be several reasons behind this, such as surgical skill, experience, familiar technique, and cleft severity. We observed that the surgeon with higher SCS incidence performed almost all SCS procedures, which in turn raise the question regarding the surgeon’s impact and threshold for SCS decision rather than surgical skill alone. Therefore, in terms of drawing definitive conclusions regarding surgical skill, more detailed studies are required, and meticulous speech assessment is needed.

## Conclusions

This study has shown that secondary palatal operations are common in children with previously repaired UCLP. Overall, outcome disparities between surgical protocols and techniques are small and derived from multiple minor factors. Despite the common challenges regarding cleft care; well-designed, prospective controlled studies with long-term follow-up are essential when assessing surgical techniques. In addition, standardized protocols for outcome measures would aid the quality of comparison between studies. Here, although no protocol proved superior to another from a long-term perspective, favorable outcomes emerged for the single-stage protocol, particularly Bardach’s 2-flap technique, with low SCS and fistula repair rates prior to ABG.
